# Mental healthcare clinician engagement with point of care testing; a qualitative study

**DOI:** 10.1186/s12888-021-03067-8

**Published:** 2021-02-04

**Authors:** Joseph Butler, Simone de Cassan, Phil Turner, Belinda Lennox, Gail Hayward, Margaret Glogowska

**Affiliations:** 1 Foundation Year 3 Physical Health Care, Department of Psychiatry, University of Oxford, Oxford, UK; 2 Core trainee Psychiatry, Oxford Health NHS Foundation Trust, Oxford, UK; 3 NIHR Community Healthcare MIC, Nuffield Department of Primary Care Health Sciences, University of Oxford, Oxford, UK; 4 Oxford Health NHS Foundation Trust, UK and Professor of Psychiatry, Department of Psychiatry, University of Oxford, Oxford, UK; 5 Nuffield Department of Primary Care Health Sciences, University of Oxford, Oxford, UK

**Keywords:** Point of care testing, POCT, Point of care device, Physical health, Severe mental illness, Community mental health team, CMHT

## Abstract

**Background:**

Point of Care Testing (POCT) is being increasingly used to augment the delivery of physical health care in a variety of settings, but their use in mental health has been limited. Research into understanding the barriers faced for successful implementation of POCT in these settings is lacking. We aimed to identify factors affecting engagement and implementation of POCT within mental health teams by exploring the attitudes to POCT, and the perceived impact POCT has on the practice of mental healthcare clinicians.

**Methods:**

Alongside a study evaluating the impact of a point of care device in Community Mental Health Teams (CMHTs), qualitative interviews were carried out with CMHT clinicians using POCT as part of annual physical checks for patients with severe and enduring mental illness. Data were collected using semi-structured interviews and analysed using thematic analysis.

**Results:**

Fifteen clinicians were interviewed across a range of professional backgrounds. Clinicians identified usability of the technology, positive impact on their patient’s experience and improved self-efficacy as drivers for successful implementation of POCT into their clinical practice. Issues with device functioning and the potential for a negative effect on the therapeutic relationship with their patients were identified as barriers. Level of physical heath training was not found to be a barrier by mental health professionals to using POCT.

**Conclusions:**

Understanding barriers and drivers for engagement is important to allow co-production of POCT and guidelines to facilitate introduction of POCT into routine clinical practice.

## Background

Point of Care Testing (POCT) is performed at or near the site of the patient, typically using a fingerprick capillary blood sample and provides rapid, actionable results [1]. Their use is becoming more widespread [2] and there is a growing body of evidence for potential benefits of POCT on patient care [3–5], clinician decision making [6, 7] and healthcare cost effectiveness [8].

Despite these advantages, POCT is not always successfully implemented into clinical teams [9] and uptake has been particularly limited in mental health teams. Research into the barriers and facilitators to POCT uptake is lacking, focussing on single teams [10], clinicians that hadn’t used POCT themselves or clinicians who were mandated to use POCT by trial protocol [9] - a situation not typical of real world POCT implementation. A broader understanding of the barriers and facilitators to POCT uptake in real world situations could help inform effective implementation of these technologies into clinical teams in the future.

Within mental healthcare, access to POCT for glycated haemoglobin (HbA1c) and Lipid Panels could support clinicians assessing the cardiovascular risk of patients with severe mental illness in the community. The physical health check is a quality standard of care comprising a history, physical examination, HbA1c and Lipid Panel blood tests [11]. Completion is currently poor; only 32.3% of patients with severe mental illness receive a full check, with blood tests the component most commonly missed [12]. This may be through non-attendance of physical health or phlebotomy appointments, as psychiatry may have a non-attendance rate as high as 20% [13].

Poor physical health in those with severe mental illness is believed to be a significant contributor to the 15 to 20 year mortality gap between these patients and those without mental illness [14, 15]. POCT could reduce the number of appointments needed to achieve this screening, and facilitate blood test measurement in patients who find it difficult to access routine primary care services. However, if POCT is to help solve this unmet health need it is critical to have an understanding of how best to implement.

Alongside a study evaluating the impact of POCT on completion of the yearly physical health check in two Community Mental Health Teams (CMHTs) [16], we conducted semi-structured interviews with clinicians, exploring their attitudes to POCT, and the perceived impact it had on their practice. We aimed to identify barriers and facilitators affecting implementation and engagement with POCT.

## Methods

**Fig. 1:**
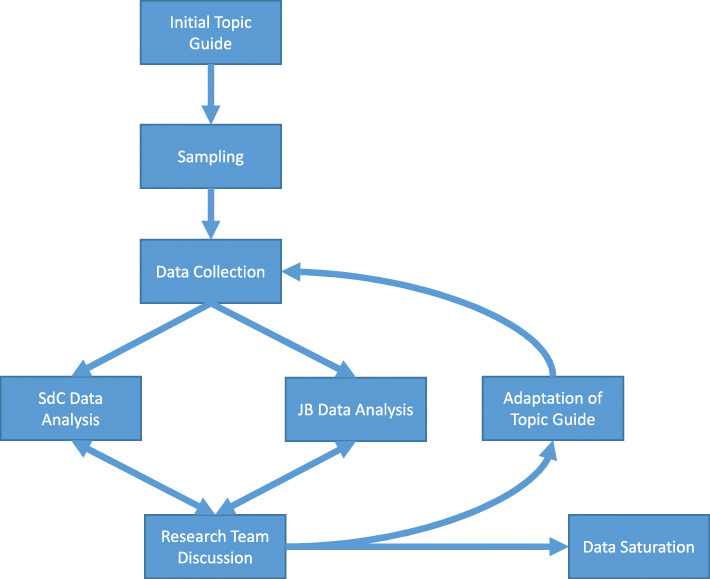
Flow chart of the qualitative research process

This article reports a qualitative clinician interview study nested within a service improvement project of a larger study looking at the impact of an ‘Afinion’ POCT device in two CMHTs [16]. CMHTs were chosen as (in contrast to inpatient services) they rely predominantly on General Practice for phlebotomy and POCT would therefore be of more use. Purposeful sampling was used to allow the research team to select the most productive sample of participants who can provide insight into the phenomenon under investigation. The study process is summarised in Fig. 1**.**

### Ethical approval

Ethical Approval was prospectively provided by the sub-committee of Wales Research Ethics Committee 6 (Reference: 18/WA/0302).

### Eligibility and recruitment

Clinicians who had access to Lipid and HbA1c POCT testing for home visits and outpatient clinics as part of their role with the Early Intervention in Psychosis Service (EIS) or the South Oxfordshire Adult Mental Health Team were included in the study. Clinicians were approached by the research team, and given an information leaflet about taking part in the interview study. All clinicians gave informed consent for participation in the study and for publication of the project findings and written quotations prior to participation. This consent was either taken in person through a signature on a consent form or via the phone after the consent form was read out to the participant and signed by the clinician. In these cases a paper copy of the consent form was sent to the participants.

### Data collection

Semi-structured interviews were conducted by JB (male) and SdC (female) using a topic guide to explore perceptions and experiences of POCT. The guide was developed initially from the literature and the experience of our research team. The guide was not pilot-tested to ensure all interviews from the limited group of participants were used, but was adapted during the interviews and adapted as topics evolved and emerged. JB and SdC were clinical academics with a research interest in evaluating POCT, but were not clinically involved in patient care or clinician use of the POCT device.

Fifteen clinician interviews were carried out, both face-to-face (11) and telephonically (4) which lasted up to 40 min. During the interviews only the participant and interviewer were present Audio-recordings were independently transcribed verbatim and analysed thematically.

### Data analysis

The transcripts were analysed thematically to identify and explore themes. The constant comparison method was utilised to ensure that all of the data were comprehensively explored and included in the analysis [17]. Researchers read and familiarised themselves with the transcripts, noting and recording initial themes, then conducted systematic and detailed open coding using *QSR NVivo 11.* An initial coding framework was derived from the topic guide and refined after initial double coding of transcripts by SdC and JB as well as discussions amongst the whole research team. Subsequent coding and analyses were completed by SdC and JB, with further group discussion to resolve differences and combine or remove codes where appropriate. The research team took an iterative stance taking forward early analysis into ongoing data collection allowing for the inclusion of emerging categories from the data ensuring themes and concepts were grounded in the data. Interviews continued until no new themes were emerging and there was sufficient explanation of those themes.

## Results

Fifteen clinicians were interviewed, seven male and eight female. Four clinicians came from the CMHT, 11 came from the EIS. Clinician backgrounds included Psychiatry (*n*=3), Nursing (*n*=6), and other allied health care professionals (*n*= 6). Due to the small number of professionals working at each study site the minimum number of demographic data was collected to ensure participant confidentiality. There were no dropouts during the study and only 1 participant declined to participate after being given information about the study. Qualitative analysis of interview transcripts highlighted two main themes, illustrated in Table 1.

**Table 1:** Table of Themes and Sub-themes

Themes	Sub-Themes
1. Engagement with POCT	a) Why Clinicians use POCT b) Usability of POCT c) The future of POCT in psychiatry
2. Perceptions of Impact of POCT on Clinicians and Patients	a) Improving Patient Experience b) Disrupting Patient Experience c) Uncovering and Communicating Abnormality d) Change in Clinician’s Role

### Engagement with POCT

#### Why clinicians use POCT

Differing opinions on whether to use POCT or not emerged during interviews. Initial engagement seemed to be affected by the anxiety of learning a new skill and fitting it in to the workload;

*“I was a bit anxious to begin with; the thought of stabbing someone with a needle”.* (C006, Allied Health)

Some clinicians were cynical and questioned whether the device’s introduction was worthwhile;

*“Is it really all about just about meeting targets again?”* (C001, Nursing)

Other clinicians saw potential in POCT, as it might be able to improve a neglected aspect of patient care, whilst others relished the opportunity to learn a new skill and develop professionally:

*“*[The POCT device] *seemed to be specifically designed to collect those two pieces of information* [Lipid panel and HbA1c] *at the same time … To me, that seemed like it was an answer to everything I’d been asking for.”* (C011, Nursing)

*“It’s important for me to be able to do this myself, and that that would give me responsibility; it would also ensure that in all areas of what they need I can provide for that. So, it was being a more complete practitioner that was really the driver for me...”* (C007, Allied Health)

#### Usability of POCT

The ease of use of the device influenced ongoing clinician engagement with POCT. Initially, interviewees reported the pressure of remembering the new information that needed to be recalled when they first tried the device ‘in the field’. Most reported the intense focus on the procedural sequence necessary for correct performance:

*“I tried to remember exactly all the routines that there were … the sequence, trying to reassure the service user … So, it was about trying to reassure her and myself, and just focus on what it was that I was trying to do and do it in such a way that it was going to be first time right.”* (C007, Allied Health)

Most clinicians were able to successfully incorporate the device into their work, citing its ease of use:

*“Yeah, that wasn’t too tricky, no. And the cartridges can only go in one way, so it wasn’t like rocket science.”* (C010, Allied Health)

The device’s speed in delivering a result was highlighted:

*“Because of the amount of time that I will spend chasing results, I’ll just go and do it myself* [on the POC device] *and get the results there and then. So it’s making my work a lot easier.”* (C013, Nursing)

There were a number of technology-related barriers identified to ongoing engagement. For nearly all the interviewees, these barriers were not deleterious enough to stop them using the device. The most frequent issue was the bulkiness of the device and the subsequent difficulties of transporting:

*“When you have got that physical health bag (and device), it is a lot to carry.”* (C002, Nursing)

The Hba1c cartridge required 1.5 μl of blood, whilst the lipid panel required ten times as much- giving more opportunity for procedural errors (insufficient sample, and air bubbles within the collecting pipette). This was summed up by one clinician:

“*… if it was going to go wrong it would go wrong with the lipids”* (C006, Allied Health)

Other barriers to using the device were identified, such as the difficulty in setting up the device in patient’s homes, the multistep sampling process being *“fiddly”*, having to let the test cartridges come to room temperature before use and not wanting to “*damage”* the POC device. It was also noted that if a patient was already undergoing routine phlebotomy (such as those prescribed Clozapine requiring regular full blood count checks), then POCT testing might result in unnecessary duplication of work.

Clinicians described a process of skill acquisition, finding their competence increase and barriers diminish as they increasingly utilised the device.

*“… the more you do it the more you kind of come across the different things that will happen and the more confident you are in just reassuring them but also still getting a result.”* (C002, Nursing)

#### The future of POCT in psychiatry

Participating clinicians were confident that POCT would feature in the future of mental healthcare provision:

*“I think it’s the way forward; it’s what we should be doing, and we should be doing it with all different types of test.”* (C009, Psychiatric)

The majority of clinicians interviewed wished to continue using POCT. Amongst this group, there were many suggestions to help facilitate further integration. Many commented that having only one device was restrictive, as only one clinician could use it at a time and in one location. Others wished for a wider repertoire of tests:

*“… if it could be used for other things as well- like full blood count, renal function – if you could measure those things it would be amazing. For those refusing bloods, and you could take those bloods, it’d be so much easier and better for everybody.”* (C014, Psychiatric)

It was suggested that the availability of a wider range of tests could promote better cooperation between Primary Care and Secondary Care Mental Health Services:

*“I would see it in the long term as taking some pressure off the GP services and enabling us to work with them more”* (C003, Allied Health)

### Perceptions of impact of POCT on clinicians and patients

POCT implementation meant clinicians utilised new skills and bore new responsibilities in assessing and managing physical health. Clinicians reflected on the impact they felt this had on the therapeutic relationship and the change in their own role.

#### Improving patient experience

Some clinicians felt their skill level increase, and enjoyed being able to offer what they felt was a better service to their patients:

*“The more skills and the wider they are, should give that service user more confidence in that* [clinician]*.”* (C007, Allied Health)

They also felt a better service included being able to offer less invasive blood sampling, which encouraged patients who wouldn’t have accepted traditional phlebotomy:

*“… she’d been in the team for a long time and she would never want to get her bloods done … she’s always refused … and then she said, ‘OK, yeh, I’ll have it done,’ And so, for her to actually have that change of attitude was brilliant.”* (C006, Allied Health)

#### Disrupting patient experience

Appearing professional in front of the patient was sometimes at odds with learning how to use a POCT device, where clinicians may make errors and perceive themselves as incompetent:

*“It’s getting the sequence … which is not very easy. And it looks bad in front of the patient”* (C001, Nursing)

*“… it just seemed a little bit unprofessional when I was in somebody’s house that was quite willing to participate, and then I couldn’t complete the whole thing.”* (C010, Allied Health)

Although errors and mistakes were initially common, the disruption to consultation flow they caused sometimes led to clinicians abandoning using the device:

*“… I didn’t know how to correct what I’d done wrong, so I didn’t want to put them through the same situation again.”* (C004, Nursing)

Some clinicians reported how POCT disrupted their normal way of care coordinating:

*“… sometimes once I’ve just pricked a patient and they’re talking and you get side-tracked with talking to them and obviously, it needs to go into a test capsule and straight into a machine for it to be a valid test.”* (C003, Allied Health)

#### Uncovering and communicating abnormality

Clinicians reflected on the advantages of early identification of metabolic pathology, especially when POC hastened detection compared to traditional care pathways:

*“… it kind of reminded me why it is important to do it and it’s not just a tick box exercise and you know we are … we’re doing it, because these people that … particularly this client as well, didn’t , you know, didn’t have any obvious risk factors in terms of diabetes.”* (C002, Nursing)

After abnormal results, clinicians would have to explain these to the client and jointly decide on a management plan. POCT was able to improve clinicians’ understanding of their patient’s physical health and helped them communicate results:

*“It was easier to then put their responses to the questions together with the blood results. And then the patient could see how the diet and lifestyle choices could be impacting on the results they got from the blood.”* (C002, Nursing)

Clinicians sometimes felt that management plans delivered with the aid of POCT were more meaningful to patients and more likely to be followed:

*“‘This is what diabetic … from forty eight upward you know; you’re forty seven; just one.’ And it looks clear, and it look … I know, I said, ‘It’s verified, and I’m sorry I’m bearing you the bad news, but this is wat you need to do.’ … And he was onboard with it.”* (C013, Nursing)

Clinicians sometimes expressed exasperation at knowing their patients’ high HbA1cs, but being unable to encourage them to improve it:

*“I’m more concerned about their health knowing those numbers … I have one patient with forty one and the other is something like forty two. They’re absolutely refusing to do any exercise and can say its my choice...”* (C001, Nursing)

#### Change in Clinician’s role

Clinicians enjoyed the increased autonomy and control that POCT gave them, which made their job easier:

*“Personally, I think it makes my work easier, because of the amount of time that I will spend chasing results. I’ll just go and do it myself and get the result there and then. So it’s making my work a lot easier.”* (C013, Nursing)

Clinicians also enjoyed the increasing diversity POCT testing brought to the role:

*… you know, it’s a fun little different thing to do.”* (C005, Nursing)

Clinicians felt engaging with POCT was able to increase their knowledge of physical health, and reflected that this knowledge was important to have.

*“So, that was really useful to know; important. I guess I need to be more aware of lipids and cholesterol and triglycerides and that sort of thing.”* (C001, Nursing)

Where clinicians were already engaged with physical healthcare, POC helped to ameliorate barriers associated with traditional care pathways, such as lack of opportunity to practice skills:

*“I did do the training for having a blood test … but I found the opportunities to practise doing it were quite few and far between.”* (C011, Nursing)

POCT was able to increase the meaningful output clinicians obtained from their work, especially when it helped clinicians overcome traditional barriers to providing physical healthcare:

“*I’ve seen results … I can see like it’s really helpful for* [patients]*; it’s really important that we know that they’re not at risk of all these disease, metabolic disease. So, we’re doing something about it, and something that I do believe in really.”* (C013, Nursing)

The POCT device was also felt to be effective in increasing access to those who wished to provide physical health checks, but who previously were not confident in doing so:

*“… it’s not just nurses now. The OT’s and the social workers are doing* [physical health] *clinics too because it’s accessible to everyone.”* (C008, Nursing)

Being able to effectively carry out a physical health check often surprised clinicians with a non-clinical background, who believed there were insurmountable barriers to integrating physical healthcare into their practice:

*“… it was clear to me that that was going to be easier to achieve if you’re a* [nurse] *or possibly an* [occupational therapist]*. But it would represent quite a shift for a social worker to be comfortable and able to do that …* [POC] *certainly has helped in as much as the kind of imaginary wall or difficulties that I may have thought there were about collecting this information, have been demolished wholesomely.”* (C007, Allied Health)

## Discussion

### Main findings

We explored Clinician attitudes following the implementation of POCT to assess cardiovascular risk in two CMHTs, seeking a wide explanation of POCT engagement. During this process, we identified barriers and facilitators to successful POCT implementation. POCT was facilitated when clinicians found the device easy to use, and when users saw benefit from their effort; when POCT improved the patient’s experience, made their job easier or improved their sense of self-efficacy. Barriers occurred when clinicians experienced errors that prevented the device’s proper functioning and when POCT had a negative impact upon the therapeutic relationship and clinicians’ perceived self-efficacy. Contrary to many participants’ expectations, a lack of formal physical health training was not a barrier to utilising POCT to monitor patients’ physical health.

### Strengths and limitations

Interviews were conducted across all professional clinician groups that make up CMHTs and also across two types of team; an early intervention in psychosis service and a more traditional CMHT, where uptake of tests differed. This allowed us to gather a broad range of perspectives on the intervention. We have identified issues that promote and inhibit uptake which are likely to be common with other novel technologies in this clinical setting.

There are limitations to the study. Most of the results seen by clinicians were normal as the caseload was predominantly within 3 years of their first acute psychotic episode. Attitudes among clinicians may have differed when more results were abnormal and might require formulating and conveying more complex management plans. The interviews took place over the intervention period where clinicians were adjusting to using the POCT device. Views may have altered after the device had become completely established within the team.

JB and SdC were working on the same site as the Early Intervention Team and supported both teams in the implementation of the device, allowing the development of a professional relationship between the researchers and participants. This could have improved rapport with the clinicians, allowing them to be more open, or it might have made negative feedback less likely. Although data collection continued until saturation, there were clinicians who were not interviewed or declined who may have held other views.

It is also vital to explore the attitudes of service users when assessing barriers and facilitators to integration of POCT and views on the provision of physical health services more broadly. These attitudes were also explored as part of the project and are presented elsewhere [16].

### Comparison with existing literature

To our knowledge, this is the first qualitative evaluation of experiences of a POCT device by clinical staff in a mental health team. Previous research has looked at POCT in the community setting, mostly in primary care to facilitate decisions of antibiotic prescription [18] or hospital admission [9] Clinicians in these studies highlight the advantages these devices can bring, and the importance of practice in allowing users to become more comfortable using the device. These themes were also expressed by clinicians in our sample.

Jones and colleagues [10] showed that teamwork, communication and trust were vital in the normalisation of POCT within the healthcare team. They also showed that perceived value in POCT helped overcome implementation barriers. This might help explain lack of uptake in some clinicians. Those who saw less value in the device did not overcome the implementation barriers and this became embedded in the culture of the team. The situation was reversed in high-uptake users; clinicians saw value, used the device more and POCT became normalised.

Our study evaluated POCT in a different clinical context. Participants were not choosing whether their client should receive the test based on the clinical situation; instead receiving the test was a care standard that should be offered to all patients. In addition, most clinicians did not have any prior experience in interpreting the results of lipid panel or HbA1c, whilst those in primary care and ambulatory care units were carrying out POCT versions of tests they were often already familiar with.

## Conclusions

Data from our sample suggests successful implementation of POCT can be facilitated in several ways. Firstly, devices must be easy-to-use, with a focus on reducing opportunity for human error, and should be transportable. Secondly training sessions should be used to increase clinician confidence in using POCT as well as how to use the results from the POCTs to formulate management plans and how to communicate these to patients effectively. Clinicians cited their patient’s perceptions of them as an important factor in the therapeutic relationship. Further research needs to address these areas in more detail and should focus on co-production of technologies and guidelines with clinicians who will ultimately utilise these tests in their practice. This would allow the local results from this study to be extrapolated to other areas of psychiatry and healthcare more broadly.

## Data Availability

Study data are transcripts of interviews containing identifiable information. Data is not publically available due to concerns participant privacy may be compromised. Anonymised and de-identified data may be requested from corresponding author.

## Abbreviations

POCT: Point of care testing
CMHT: Community mental health team
EIS: Early Intervention in psychosis service
